# Feasibility and safety of the direct current cardioversion at the time of left atrial appendage occlusion for patients with atrial fibrillation

**DOI:** 10.3389/fcvm.2023.1219611

**Published:** 2023-09-08

**Authors:** Xian Sai Meng, Tao Chen, Xin Yan Wang, Xu Lu, Jia Hu, Juan Shen, Jun Guo

**Affiliations:** Department of Cardiovascular, The Sixth Medical Center of Chinese PLA General Hospital, Beijing, China

**Keywords:** atrial fibrillation, left atrial appendage, direct current cardioversion, left atrial appendage occlusion, stroke

## Abstract

**Background:**

With an increasing number of patients undergoing left atrial appendage occlusion (LAAO), more attention is being paid to relieving clinical symptoms and improving the quality of life of these patients. For patients with atrial fibrillation (AF), direct current cardioversion (DCCV) is an alternate, nonpharmacological choice to restore sinus rhythm and relieve clinical symptoms.

**Objectives:**

The purpose of this study was to assess the feasibility and safety of the DCCV at the time of LAAO for patients with AF.

**Methods:**

Forty patients were enrolled in the DCCV group undergoing the DCCV at the time of LAAO. The control group undergoing LAAO alone was formed by 1:1 matching.

**Results:**

In the DCCV group, cardioversion was immediately successful in 30 (75%) patients, of which 12 (40%) had AF recurrence at the three-month follow-up. The failed-DCCV group was older (73.70 ± 4.74 vs. 62.20 ± 9.01 years old, *P* = 0.000), had a faster postcardioversion heart rate (88.80 ± 16.58 vs. 70.97 ± 14.73 times, *P* = 0.03), and had a higher mean HAS-BLED score (4.00 vs. 3.00, *P* = 0.01) than the successful-DCCV group. No patients experienced periprocedural pericardial effusion, occluder displacement, device embolism, or >5 mm peridevice leakage. One patient experienced a transient ischemic attack (TIA) in the DCCV group during the follow-up.

**Conclusions:**

The DCCV at the time of LAAO is feasible and safe for AF patients with contraindications for catheter ablation or AF recurrence after previous catheter ablation to restore the sinus rhythm and relieve clinical symptoms. The DCCV at the time of LAAO is more likely to succeed for younger patients and patients with lower HAS-BLED scores.

## 1. Introduction

Although anticoagulant therapy is the primary treatment for atrial fibrillation (AF), its benefits do not clearly outweigh the risks for patients with a high bleeding risk. Therefore, left atrial appendage occlusion (LAAO) has become the main alternative for stroke prevention in patients with high HAS-BLED scores for nonvalvular AF, in whom 90% of the thrombi originate from the left atrial appendage (LAA) (1–5). With an increasing number of patients undergoing LAAO, more attention is being paid to relieving their clinical symptoms and improving their quality of life. For patients in whom catheter ablation is a contraindication or who have experienced AF recurrence after previous catheter ablation, DCCV is an alternate, nonpharmacological choice to restore sinus rhythm and relieve clinical symptoms (6, 7). It is theoretically feasible to perform the DCCV at the time of LAAO for AF patients. Previous studies have evaluated the safety and feasibility of DCCV for patients with LAAO devices after implantation (8). However, there have been no studies on the DCCV at the time of LAAO for AF patients. Therefore, the objective of this study was to assess the feasibility and safety of the DCCV at the time of LAAO for AF patients.

## 2. Methods

### 2.1. Study population

Patients with AF who underwent LAAO at the Chinese People's Liberation Army General Hospital (Beijing, China) between October 2017 and September 2021 were enrolled in this study. The study retrospectively involved 80 patients with WATCHMAN^TM^ (Boston Scientific, MA, USA) devices, including the DCCV group and the control group, who were matched using propensity score matching by procedure month, age and type of AF with a 1:1 proportion. The inclusion criteria of the DCCV group in the current study were as follows: (і) age > 18 years, (ii) catheter ablation as a contraindications or previous AF recurrence after catheter ablation, (iii) The DCCV at the time of LAAO, and (iv) long-term follow-up (three- and six-month visits). The subjects who had contraindications to DCCV and underwent combined LAAO and catheter ablation were excluded. Forty patients undergoing LAAO alone were enrolled in the control group. This study was approved by the Medical Ethics Committee of the Chinese People's Liberation Army General Hospital and complied with the principles of the Declaration of Helsinki. All patients provided informed consent before participating in this study.

### 2.2. Baseline data and observation index

Baseline and periprocedural electrocardiograms (ECGs) were performed to record the heart rate and rhythm of the heart. Transthoracic echocardiography (TTE), multislice computed tomography (MSCT), and transesophageal echocardiography (TEE) were performed to evaluate cardiac function, measure the structural parameters of the left atrium (LA), and exclude the presence of intracardiac thrombi. We focused on the success and recurrence rates of DCCV, as well as of adverse events, during the follow-up period to explore the feasibility of periprocedural DCCV. In addition, patients' demographics, AF type, medical history, heart rate, left atrial size, and medication regimens were analyzed as factors related to DCCV failure and recurrence of AF after cardioversion. Meanwhile, we explore predictive factors for DCCV failure and recurrence of AF after cardioversion. The feasibility observation index was the success rate of cardioversion, and the safety observation index was postprocedural adverse event.

### 2.3. Direct current cardioversion

Prior to the commencement of the procedure, a TEE was conducted to assess the LAA and exclude the presence of LAA thrombosis. After deep sedation, DCCV is performed before or after the release of the occluder, and using synchronous electrical biphasic current (75 to 360 J) determined by the physician. Anterior wall electrode pad placed on the anterior thoracic wall, typically at the right midclavicular line and the fourth intercostal space; posterior wall the patch placed on the posterior aspect of the thoracic wall, usually between the left scapular angle and the spine. Cardioversion failure was defined as AF remaining unconverted after three consecutive cardioversions. We defined AF as successful cardioversion if it did not recur within 15 min after DCCV. Subjects with DCCV were grouped into failed-DCCV and successful DCCV groups, depending on DCCV outcome.

### 2.4. Left atrial appendage occlusion

LAAO was conducted according to previously published procedures (9, 10). The whole procedure was performed under the guidance of the TEE and fluoroscopy. Following the establishment of a peripheral venous pathway, utilizing TEE guidance, proceed to puncture the atrial septum and implant the occluder. The compression ratio of the occluder was 8%–20% to ensure the stability of the occluder after LAAO (11, 12). Conclusively assess the occluder status through the utilization of TEE and fluoroscopy.

The occluder meet the PASS (position, anchor, size, and sealing) criteria.

### 2.5. Antithrombotic strategy after LAAO and follow-up

The patients began receiving dabigatran 110 mg twice daily or rivaroxaban 15 mg once daily after the procedure. At the three-month follow-up after the procedure, if there was no device-related thrombosis (DRT) and the peridevice leak (PDL) was <5 mm, the antithrombotic strategy was adjusted to 100 mg aspirin once daily plus 75 mg clopidogrel once daily. If there was DRT or PDL > 5 mm, the antithrombotic strategy was continued until the next follow-up. At the six-month follow-up, aspirin will continue to be administered to the patient.

Follow-up was conducted by clinical visits combined with telephone follow-up at three- and six-month after the procedure. Follow-up visits at three and six months postoperatively included ECG, TTE, and MSCT. Patient rhythm state and occluder location were assessed, and postoperative adverse events (death, pericardial effusion, stroke, major bleeding, DRT, and PDL) were recorded. At the three-month follow-up, the patients in the successful DCCV group were divided into the nonrecurrence and the recurrence groups according to whether they had recurrent AF.

### 2.6. Statistical methods

The propensity score matching and all statistical analyses in this study were performed using SPSS 22.0. According to the distribution, continuous variables are presented as the mean ± SD or median (quartile 1–quartile 3) and were compared using the t-test or Wilcoxon test. Categorical variables are described as counts (%) and compared using Pearson's chi-square test (or Fisher's exact test). Binary logistic regression analysis was performed to assess the factors associated with failure and the recurrence of cardioversion. A *P* value < 0.05 was considered statistically significant.

## 3. Results

### 3.1. Clinical characteristics

Forty patients were enrolled in the DCCV group undergoing the DCCV at the time of LAAO. Forty matched patients were recruited for the control group undergoing LAAO alone. LAAO was successfully performed in all patients. In the DCCV group, 28/40(70%) patients had a history of catheter ablation procedures, while 11/40(27.5%) patients had undergone two or more catheter ablation treatments. The mean ages of the DCCV and control groups were 65.08 ± 9.54 and 65.13 ± 10.02 years, respectively. However, the preprocedural heart rate (87 beats vs. 75.5 beats, *P* < 0.001) and the mean left atrial volume (197.50 ± 50.39 vs. 165.57 ± 52.38 mm 3, *P* = 0.02) were significantly higher in the DCCV group than in the control group. Other baseline data showed no significant difference. The baseline characteristics of the DCCV group are shown in Table 1.

**Table 1:** Baseline characteristics and periprocedural results.

	DCCV group (*n* = 40)	Control group (*n* = 40)	*P* Value
Age (years)	65.08 ± 9.54	65.13 ± 10.02	0.98
Male *n* (%)	29 (72.5)	26 (65.0)	0.47
BMI (kg/m^2^)	26.53 ± 3.44	26.25 ± 3.52	0.72
Renal insufficiency *n* (%)	4 (10.0)	3 (7.5)	1.00
Mitral regurgitation *n* (%)			0.66
None	11 (27.5)	15 (37.5)	
Mild -moderate	26 (65.0)	23 (57.5)	
Severe	3 (7.5)	2 (5.0)	
AF type *n* (%)			1.00
Paroxysmal	15 (37.5)	15 (37.5)	
Persistent	25 (62.5)	25 (62.5)	
Pre-procedural heart rate (times)	87.00 (77.25–98.00)	75.50 (67.50–82.00)	<0.001
Post-cardioversion heart rate (times)	75.43 ± 16.91		
CHA2DS2-VASC score	4.50 (3.00–6.00)	5.00 (4.00–6.00)	0.54
HAS-BLED score	3.00 (2.25–4.00)	3.00 (2.00–4.00)	0.25
Medical history			
Cardiac insufficiency *n* (%)	15 (37.5)	9 (22.5)	0.14
Hypertension *n* (%)	30 (75.0)	30 (75.0)	1.00
Diabetes *n* (%)	13 (32.5)	13 (32.5)	1.00
TIA/ stroke *n* (%)	24 (60.0)	29 (72.5)	0.24
myocardial infarction *n* (%)	4 (10.0)	4 (10.0)	1.00
History of bleed *n* (%)	5 (12.5)	7 (17.5)	0.53
LVEF	0.58 (0.53–0.62)	0.58 (0.52–0.62)	0.99
LA anteroposterior diameter (mm)	42.78 ± 4.42	41.68 ± 4.75	0.29
LA volume (mm^3^)	197.50 ± 50.39	165.57 ± 52.38	0.02
LAA diameter (mm)	23.48 ± 3.27	23.55 ± 3.32	0.92
LAA type *n* (%)			0.65
Chicken wing	2 (5.0)	5 (12.5)	
Windsock	4 (10.0)	3 (7.5)	
Cactus	23 (57.5)	20 (50.0)	
Cauliflower	10 (25.0)	9 (22.5)	
Inverted chicken wing	1 (2.5)	3 (7.5)	
Preprocedural medication			
β-blocker *n* (%)	33 (82.5)	29 (72.5)	0.28
ACER/ARB *n* (%)	9 (22.5)	10 (25.0)	0.79
Calcium-channel blocker *n* (%)	14 (35.0)	17 (42.5)	0.49
Diuretic *n* (%)	15 (37.5)	10 (25.0)	0.23
Watchman Device size *n* (%)			0.78
24 mm	7 (17.5)	10 (25.0)	
27 mm	10 (25.0)	11 (27.5)	
30 mm	11 (27.5)	8 (20.0)	
33 mm	12 (30.0)	11 (27.5)	
Device compression rates (%)^a^	18.00 (14.33–20.98)	20.51 (17.00–23.18)	0.14
Procedure time (min)	71.28 ± 14.69	62.20 ± 6.75	0.001
PDL *n* (%)^a^^,^^b^	3 (7.5)	2 (5.0)	1.00
DCCV before the release of occluder *n* (%)	31 (77.5)		
The space of time from the release of occluder to DCCV (min)^c^	15.33 ± 6.33		
DCCV energy (J)	150 (150–200)		
Number of DCCV *n* (%)			
1 time	27 (67.5)		
≥2 times	13 (32.5)		
Successful DCCV	30 (75.0)		

LA, left atrial; LAA, left atrial appendage occlusion; PDL, peri-device leak; DCCV, direct current cardioversion.

^a^ Obtained through TEE.

^b^ All PDLs are less than 3 mm.

^c^ The space of time from the release of the occluder to DCCV in patients with DCCV after the release of the occluder.

### 3.2. Periprocedural results

The WATCHMAN™ (Boston Scientific, MA, USA) device was successfully implanted in all patients in the DCCV group. The 33 mm occluder was the most commonly employed, which was used by 12/40 (30%) of the patients. Three subjects in the DCCV group and two subjects in the control group experienced PDL (less than 3 mm) (7.5% vs. 5.0%, *P* = 1.00) as detected by TEE. In addition, no patients experienced periprocedural pericardial effusion, occluder displacement, device embolism, or >5 mm PDL. The median DCCV energy of all the patients was 150 J. Cardioversion was immediately successful in 30/40 (75%) patients. DCCV was performed in nine patients after the release of the occluder and in thirty-one patients before during the periprocedural period. The space of time from the release of the occluder to DCCV was 15.33 ± 6.33 min in patients with DCCV after the release of the occluder. Other periprocedural conditions of patients and the use of occluders are shown in Table 1.

### 3.3. Relevant factors for DCCV failure

In the DCCV group, 10/40 (25%) showed DCCV failure and did not show any significant differences in preprocedural heart rate (84.40 ± 6.45 vs. 93.00 ± 21.28, *P* = 0.31) or CHA2DS2-VASc score (5.10 ± 2.23 vs. 4.13 ± 1.87, *P* = 0.19) compared to the patients with successful DCCV. There was also no statistical difference between the two groups in the degree of mitral regurgitation (*P* = 0.07) and in the LAA type (*P* = 0.94). However, the failed-DCCV group was older than the successful-DCCV group (73.70 ± 4.74 vs. 62.20 ± 9.01 years old, *P* < 0.001). Further, the postcardioversion heart rate was significantly faster (88.80 ± 16.58 vs. 70.97 ± 14.73 times, *P* = 0.03) and the HAS-BLED score (4.00 vs. 3.00, *P* = 0.01) was significantly higher in the failed-DCCV group than in the successful DCCV group (Table 2). In addition, age, postcardioversion heart rate, and the HAS-BLED score were integrated into the binary logistic regression analysis. The results showed that age (odds ratio [OR] 1.27, 95% confidence interval [CI] 1.002–1.598, *P* = 0.048) and postcardioversion heart rate (OR 1.09, 95% CI 1.006–1.182, *P* = 0.03) were closely related to the success of DCCV.

**Table 2:** Comparison of patients with failed DCCV and successful DCCV.

	Failed DCCV (*n* = 10)	Successful DCCV (*n* = 30)	*P* Value
Age (years)	73.70 ± 4.74	62.20 ± 9.01	<0.001
Male *n* (%)	7 (70.0)	22 (73.3)	1.00
BMI (kg/m^2^)	26.65 ± 2.62	26.49 ± 3.71	0.90
Renal insufficiency *n* (%)	0 (0.0)	4 (13.3)	0.56
Mitral regurgitation *n* (%)			0.07
None	4 (40.0)	7 (23.3)	
Mild -moderate	4 (40.0)	22 (73.7)	
Severe	2 (20.0)	1 (3.3)	
Atrial fibrillation type *n* (%)			0.35
Paroxysmal	2 (20.0)	13 (43.3)	
Persistent	8 (80.0)	17 (56.7)	
Preprocedural heart rate (times)	84.40 ± 6.45	93.00 ± 21.28	0.31
Postcardioversion heart rate (times)	88.80 ± 16.58	70.97 ± 14.73	0.03
CHA2DS2-VASC score	5.10 ± 2.23	4.13 ± 1.87	0.19
HAS-BLED score	4.00 (3,5)	3.00 (2,4)	0.01
LVEF	0.58 (0.53–0.60)	0.58 (0.54–0.62)	0.79
LA anteroposterior diameter (mm)	43.40 ± 3.44	42.57 ± 4.73	0.61
LA volume (mm^3^)	194.24 ± 27.44	198.75 ± 57.32	0.83
LAA type *n* (%)			0.94
Chicken wing	0 (0.0)	2 (6.7)	
Windsock	1 (10.0)	3 (10.0)	
Cactus	7 (70.0)	16 (53.3)	
Cauliflower	2 (20.0)	8 (26.7)	
Inverted chicken wing	0 (0.0)	1 (3.3)	
DCCV before the release of occluder *n* (%)	9 (90.0)	22 (73.3)	0.51
DCCV energy (J)	174.50 ± 65.42	169.50 ± 52.79	0.70
Recurrence at 3-month follow-up *n* (%)		12 (40.0)	
Recurrence at 6-month follow-up *n* (%)		13 (43.3)	
Symptoms at 3-month follow-up *n* (%)		8 (26.7)	
Symptoms at 6-month follow-up *n* (%)		11 (36.7)	

LA, left atrial; LAA, left atrial appendage occlusion; DCCV, direct current cardioversion.

### 3.4. Follow-up

At the three-month follow-up, there was no significant difference in postprocedural antithrombotic strategies between the nonrecurrence and the recurrence groups. In the DCCV and control groups, no patients experienced occluder displacement. In the DCCV group, one patient experienced a transient ischemic attack (TIA) and had a 3 mm PDL, as shown by the MSCT. In the control group, one patient experienced a stroke, and one patient developed DRT during the follow-up. There were no significant differences in postprocedural adverse events between the DCCV and the control groups (2.5% vs. 5%, *P* = 1.00). Table 3 shows the postprocedural medication regimens and adverse events of both groups.

**Table 3:** Postprocedural medication strategy and adverse event.

	DCCV group (*n* = 40)	Control group (*n* = 40)	*P* Value
Postprocedural medication strategy			
Anticoagulants *n* (%)			0.12
Nothing	5 (12.5)	8 (20.0)	
Rivaroxaban	12 (30.0)	16 (40.0)	
Dabigatran	23 (57.5)	14 (35.0)	
Warfarin	0 (0.0)	2 (5.0)	
Antiplatelet *n* (%)			0.09
Nothing	36 (90.0)	33 (82.5)	
Single antiplatelet^a^	2 (5.0)	0 (0.0)	
Dual antiplatelet^b^	2 (5.0)	7 (17.5)	
Ⅰ and Ⅱ antiarrhythmic *n* (%)			<0.001
Nothing	18 (45.0)	38 (95.0)	
Propafenone	5 (12.5)	2 (5.0)	
Amiodarone	17 (42.5)	0 (0.0)	
β-blocker *n* (%)	28 (70.0)	28 (70.0)	1.00
Spironolactone *n* (%)	16 (40.0)	12 (30.0)	0.35
Postprocedural adverse event *n* (%)^c^	1 (2.5)	2 (5.0)	1.00

^a^ Single Antiplatelet: aspirin or clopidogrel.

^b^ Dual Antiplatelet: aspirin and clopidogrel.

^c^ One adverse event is TIA in the DCCV group and 2 adverse events in the control group are stroke and DRT during follow-up.

### 3.5. Relevant factors for the recurrence of AF after cardioversion

**Figure 1:**
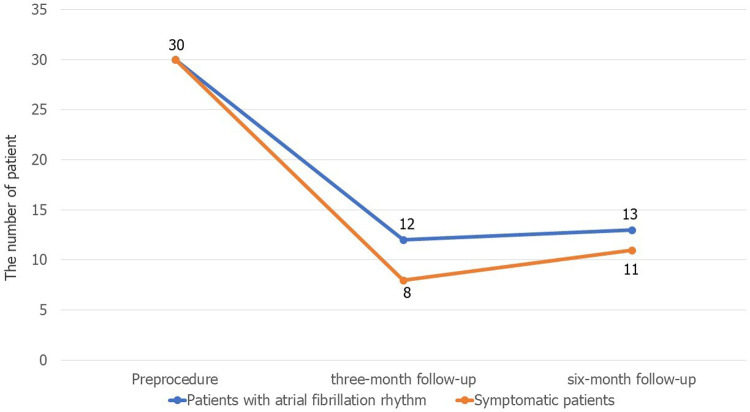
The changes in patients' symptoms..

The results of follow-up in the successful DCCV group showed that 12/30 (40%) had recurrent AF at the three-month follow-up, and the number of patients with recurrence increased to 13 (43.3%) at the six-month follow-up. Figure 1 shows the changes in the patients' symptoms. At the three-month follow-up, there were no significant differences in the renal insufficiency (5.6% vs. 25.0%, *P* = 0.27), BMI (26.42 ± 3.95 vs. 26.58 ± 3.49 kg/m^2^, *P* = 0.91) and LA anteroposterior diameter (42.33 ± 4.47 vs. 42.92 ± 5.28 mm, *P* = 0.75) or LA volume (201.84 ± 62.63 vs. 193.72 ± 51.14 mm^3^, *P* = 0.76) between the nonrecurrence and the recurrence groups. However, there were significant differences in the ratio of the anteroposterior diameter to the vertical diameter of the left atrium (0.67 ± 0.07 vs. 0.73 ± 0.06, *P* = 0.02) and the ratio of the anteroposterior diameter to the transverse diameter (0.93 ± 0.09 vs. 1.03 ± 0.13, *P* = 0.03) between the nonrecurrence and the recurrence groups. Details are shown in Table 4.

**Table 4:** Comparison of patients with AF recurrence and without recurrence after successful DCCV.

	No recurrence (*n* = 18)	Recurrence (*n* = 12)	*P* Value
Age (years)	63.39 ± 9.27	60.42 ± 7.07	0.39
Male *n* (%)	13 (72.2)	9 (75.0)	1.00
BMI (kg/m^2^)	26.42 ± 3.95	26.58 ± 3.49	0.91
Renal insufficiency *n* (%)	1 (5.6)	3 (25.0)	0.27
Mitral regurgitation *n* (%)			0.64
None	3 (16.7)	4 (33.3)	
Mild -moderate	14 (77.8)	8 (66.7)	
Severe	1 (5.6)	0 (0.0)	
Atrial fibrillation type *n* (%)			0.88
Paroxysmal	8 (44.4)	5 (41.7)	
Persistent	10 (55.6)	7 (58.3)	
CHA2DS2-VASC score	3.94 ± 1.70	4.42 ± 2.15	0.44
HAS-BLED score	3.00 (2.00–4.00)	3.00 (3.00–3.50)	0.51
LVEF	0.58 (0.55–0.61)	0.58 (0.48–0.63)	0.66
LA anteroposterior diameter (mm)	42.33 ± 4.47	42.92 ± 5.28	0.75
Ratio of anteroposterior to vertical diameter of LA	0.67 ± 0.07	0.73 ± 0.06	0.02
Ratio of anteroposterior to transverse diameter of LA	0.93 ± 0.09	1.03 ± 0.13	0.03
LA volume (mm^3^)	201.84 ± 62.63	193.72 ± 51.14	0.76
LAA type *n* (%)			0.78
Chicken Wing	1 (5.6)	1 (8.3)	
Windsock	2 (11.1)	1 (8.3)	
Cactus	9 (50.0)	7 (58.3)	
Cauliflower	6 (30.3)	2 (16.7)	
Inverted chicken wing	0 (0.0)	1 (8.3)	
Postprocedural medication strategy			
Anticoagulants *n* (%)			0.39
Nothing	1 (5.6)	1 (8.3)	
Rivaroxaban	3 (16.7)	5 (41.7)	
Dabigatran	14 (77.8)	6 (50.0)	
Antiplatelet *n* (%)			0.23
Nothing	16 (88.9)	12 (100.0)	
^ ^Single antiplatelet^a^	2 (9.5)	0 (0.0)	
^ ^Dual antiplatelet^b^	0 (0.0)	0 (0.0)	
Ⅰ and Ⅱ antiarrhythmic			0.56
Nothing *n* (%)	7 (38.9)	4 (33.3)	
Propafenone *n* (%)	4 (22.2)	1 (8.3)	
Amiodarone *n* (%)	7 (38.9)	7 (58.3)	
β-blocker *n* (%)	12 (66.7)	6 (50.0)	0.59
Spironolactone *n* (%)	7 (38.9)	6 (50.0)	0.55

LA, left atrial.

^a^ Single antiplatelet: aspirin or clopidogrel.

^b^ Dual antiplatelet: aspirin and clopidogrel.

## 4. Discussion

Our study demonstrates that the DCCV at the time of LAAO for AF patients is feasible and safe, with a success rate of 70% and a recurrence rate of 40% at the 3-month follow-up. Compared with LAAO alone, the DCCV at the time of LAAO does not increase postprocedural adverse events. In addition, the DCCV at the time of LAAO was more likely to succeed in patients with younger age and lower HAS-BLED scores. And postcardioversion heart rate was also closely related to the success of cardioversion.

### 4.1. Benefits of the DCCV at the time of LAAO for patients

In this study, the decision to perform DCCV at the time of LAAO in patients was based on the following considerations: Firstly, compared to the control group, the DCCV group had a faster preprocedural heart rate, which not only aggravates the symptoms of AF but also affected the implantation of the device. the patients' mean heart rate decreased from 87 to 75.43 beats/min after DCCV, which facilitating for the operation, as also shown previously (13). Secondly, the patients presented with contraindications to catheter ablation, including a large left atrial volume and an acute stage of cerebral infarction (6), or with recurrent AF after multiple catheter ablation. However, the patients still expressed a strong desire for the restoration of sinus rhythm. Thirdly, AF-induced rapid ventricular rate led to hemodynamic instability, necessitating urgent cardioversion. The DCCV at the time of LAAO relieved patient symptoms. Furthermore, because DCCV was performed after deep sedation, the DCCV at the time of LAAO not only did not increase the additional sedatives and patient burden, but also avoids the discomfort caused by separate DCCV (14).

### 4.2. Relevant factors for DCCV failure and recurrence of AF after cardioversion

In this study, DCCV was successful in 30 cases, with a success rate of 75%. AF recurred in 12 patients with successful DCCV in the third month after the procedure, with a recurrence rate of 40%. These success and recurrence rates of DCCV were the same as those previously reported in elderly patients with AF (15–18). Both success and recurrence rates were within the acceptable range.

The datas concerning relevant factors for DCCV failure are scarce. Kim et al. explored relevant factors for cardioversion failure and AF recurrence after successful cardioversion (19). Similar to our study, they analyzed the clinical characteristics and medication regimens of patients to identify relevant factors for DCCV failure and AF recurrence after cardioversion. In contrast to their study, our studied factors include the CHA2DS2-VASc score, HAS-BLED score, and heart rate, and DCCV was performed perioperatively. Our results showed that advanced age, faster postcardioversion ventricular rate, and higher HAS-BLED scores were closely related to the failure of the DCCV at the time of LAAO. Previous studies have confirmed that advanced age and medical history of diseases, such as hypertension and diabetes, are risk factors for AF (15, 20). This information is helpful for clinical decision making involving DCCV therapy in patients undergoing LAAO. A supervulnerable status of the atrium after DCCV might contribute to a faster heart rate and subsequent recurrence within 15 min of DCCV (21, 22).

In addition, the recurrence rate of AF is high after DCCV (23). In previous studies (24), the LA diameter was not related to cardioversion failure or AF recurrence. In line with these findings, our results of the analysis of 30 patients with successful DCCV suggest that there is no significant correlation between the LA diameter and AF recurrence after DCCV. Interestingly, we found that the ratio of the anteroposterior diameter to the vertical diameter of the LA and the ratio of the anteroposterior diameter to the transverse diameter of the LA were greater in the recurrence group than in the nonrecurrence group in this study. This suggests that for patients presenting with a higher the ratio of the anteroposterior diameter to the vertical or transverse diameter of the LA, the decision regarding DCCV at the time of LAAO requires thorough and meticulous consideration.

### 4.3. Safety of the DCCV at the time of LAAO

In our study, there were 9 patients who were treated with DCCV after the release of the occluder. In theory, for patients with implanted occlusion devices, the DCCV will impact the heart and the occluder, which might create the risk of displacement or even embolization of the occluder. However, there are no relevant reports of such events. Hanazawa et al. reported a single case of stroke on the second day after DCCV (25). The cause of stroke might be the incomplete evaluation of the LAA on preprocedural imaging. Sharma et al. conducted the first retrospective study (8) to demonstrate the safety of TEE-guided DCCV in patients with LAAO. In our study, there was one TIA in the DCCV group and two adverse events (stroke, DRT) in the control group during follow-up. In the DCCV group, DCCV of patients with TIA was performed before the release of the occluder. MSCT showed that the occluder was well-positioned, and there was a 3 mm PDL but no evidence of DRT. In addition, despite 1 patient in the DCCV group receiving single antiplatelet therapy and 1 patient not receiving any antithrombotic therapy, and one patient in the control group not receiving any antithrombotic therapy following the LAAO (26), no bleeding events occurred during the follow-up period. The incidence of postoperative adverse events was not higher in the DCCV group than in the control group. Therefore, the DCCV at the time of LAAO is safe and does not increase the incidence of adverse events after the procedure.

### 4.4. Study limitations

First, the retrospective nature was an inherent limitation and selection bias cannot be ruled out in patients with DCCV. Second, the small sample size was the biggest limitation of this study. Although the results of our study showed that there was no increase in adverse events after the DCCV at the time of LAAO, in-depth prospective studies with large samples and multiple centers are needed to show the safety of this procedure. Third, we need to further explore whether DCCV has an impact on occluder position adjustment, release, and rivets during the procedure.

## 5. Conclusions

The DCCV at the time of LAAO is feasible and safe for AF patients with contraindications for catheter ablation or AF recurrence after previous catheter ablation to restore the sinus rhythm and relieve clinical symptoms. The DCCV at the time of LAAO is more likely to succeed for younger patients and patients with lower HAS-BLED scores.

## Data Availability

The original contributions presented in the study are included in the article/Supplementary Material, further inquiries can be directed to the corresponding author.
